# Heme oxygenase-independent bilin biosynthesis revealed by a *hmox1* suppressor screening in *Chlamydomonas reinhardtii*

**DOI:** 10.3389/fmicb.2022.956554

**Published:** 2022-08-08

**Authors:** Weiqing Zhang, Rui Deng, Weida Shi, Zheng Li, Robert M. Larkin, Qiuling Fan, Deqiang Duanmu

**Affiliations:** ^1^State Key Laboratory of Agricultural Microbiology, College of Life Science and Technology, Shenzhen Institute of Nutrition and Health, Huazhong Agricultural University, Wuhan, China; ^2^Key Laboratory of Horticultural Plant Biology, Ministry of Education, Huazhong Agricultural University, Wuhan, China; ^3^Shenzhen Branch, Guangdong Laboratory for Lingnan Modern Agriculture, Genome Analysis Laboratory of the Ministry of Agriculture, Agricultural Genomics Institute at Shenzhen, Chinese Academy of Agricultural Sciences, Shenzhen, China

**Keywords:** heme oxygenase, heme, bilin, suppressor screening, insertional mutagenesis

## Abstract

Bilins are open-chain tetrapyrroles synthesized in phototrophs by successive enzymic reactions catalyzed by heme oxygenases (HMOXs/HOs) and ferredoxin-dependent biliverdin reductases (FDBRs) that typically serve as chromophore cofactors for phytochromes and phycobiliproteins. *Chlamydomonas reinhardtii* lacks both phycobiliproteins and phytochromes. Nonetheless, the activity and stability of photosystem I (PSI) and the catalytic subunit of magnesium chelatase (MgCh) named CHLH1 are significantly reduced and phototropic growth is significantly attenuated in a *hmox1* mutant that is deficient in bilin biosynthesis. Consistent with these findings, previous studies on *hmox1* uncovered an essential role for bilins in chloroplast retrograde signaling, maintenance of a functional photosynthetic apparatus, and the direct regulation of chlorophyll biosynthesis. In this study, we generated and screened a collection of insertional mutants in a *hmox1* genetic background for suppressor mutants with phototropic growth restored to rates observed in wild-type 4A+ *C. reinhardtii* cells. Here, we characterized a suppressor of *hmox1* named *ho1su1* with phototrophic growth rates and levels of CHLH1 and PSI proteins similar to 4A+. Tetrad analysis indicated that a plasmid insertion co-segregated with the suppressor phenotype of *ho1su1*. Results from TAIL-PCR and plasmid rescue experiments demonstrated that the plasmid insertion was located in exon 1 of the *HMOX1* locus. Heterologous expression of the bilin-binding reporter *Nostoc punctiforme* NpF2164g5 in the chloroplast of *ho1su1* indicated that bilin accumulated in the chloroplast of *ho1su1* despite the absence of the HMOX1 protein. Collectively, our study reveals the presence of an alternative bilin biosynthetic pathway independent of HMOX1 in the chloroplasts of Chlamydomonas cells.

## Introduction

The chloroplast is a vital organelle in photosynthetic eukaryotes that performs photosynthesis and synthesizes important metabolites such as fatty acids, vitamins, amino acids, and tetrapyrroles (Jarvis and López-Juez, 2013). The nucleus uses the process of anterograde signaling to precisely control the gene expression, and the structure and function of chloroplasts (Berry et al., 2013; Singh et al., 2015). Meanwhile, the chloroplast is frequently confronted with endogenous developmental cues and environmental challenges (Li and Kim, 2021; Woodson, 2022). The chloroplast communicates its functional status to the nucleus by emitting signals, namely chloroplast retrograde signals. These signals regulate the expression of nuclear genes that are essential for chloroplast biogenesis, maintenance, and stress responses (Chan et al., 2016; Mielecki et al., 2020). The molecular mechanism of chloroplast retrograde signaling was extensively characterized through the study of *genomes uncoupled* (*gun*) mutants in *Arabidopsis thaliana* (Susek et al., 1993; Larkin, 2014). Among six identified *GUN* genes, *GUN2*-*GUN6* encode enzymes and regulatory proteins involved in tetrapyrrole biosynthesis, indicating the important roles of tetrapyrrole intermediates as chloroplast retrograde signals (Susek et al., 1993; Wu and Bock, 2021).

Tetrapyrroles are a class of cyclic and linear molecules containing four pyrrole rings that are widely distributed in animals, plants, and microbes. Tetrapyrroles commonly serve as cofactors for proteins involved in important biological processes, such as photosynthesis and respiration (Tanaka and Tanaka, 2007; Mochizuki et al., 2010). Tetrapyrroles are made from glutamate in the plastid of photosynthetic eukaryotes and are converted to 5-aminolevulinic acid (ALA)—the universal precursor of tetrapyrroles—and are further converted to protoporphyrin IX (PPIX) (Tanaka et al., 2011; Brzezowski et al., 2015). Magnesium chelatase (MgCh) synthesizes Mg-PPIX from PPIX by inserting a Mg^2+^ ion into PPIX, which is a precursor for chlorophyll. Ferrochelatase (FeCh) synthesizes heme from PPIX by inserting a Fe^2+^ ion into PPIX (Busch and Montgomery, 2015). Heme oxygenases (HMOXs/HOs) convert heme into biliverdin IXα (BV), which is subsequently reduced to various types of bilins by a class of ferredoxin-dependent biliverdin reductases (FDBRs) with regiospecificities for BV reduction in phototrophs (Nagahatenna et al., 2015; Rockwell and Lagarias, 2017).

Bilins generally serve as cofactors for phytochromes and phycobiliproteins, which contribute to light perception and light harvesting in oxygenic phototrophs (Adir et al., 2020; Fushimi and Narikawa, 2021). Despite the absence of both phytochromes and phycobiliproteins, the model photosynthetic organism *Chlamydomonas reinhardtii* contains HMOX1 and PCYA1 (phycocyanobilin ferredoxin oxidoreductase) that enable phycocyanobilin (PCB) production in the chloroplast (Duanmu et al., 2013; Rockwell et al., 2014). Previous studies on a Chlamydomonas *hmox1* mutant revealed that bilin is a promising chloroplast retrograde signal that regulates the expression of genes associated with the detoxification of reactive oxygen species (ROS) that are essential to alleviate oxidative stress during the diurnal dark-to-light transition (Duanmu et al., 2013, 2017). Bilin also functions in the chloroplast to maintain the stability of photosystem I (PSI) and to sustain a robust photosynthetic apparatus during photo-acclimation (Wittkopp et al., 2017). Additionally, bilin regulates chlorophyll biosynthesis in *C. reinhardtii* by promoting the activity of MgCh and the stability of the catalytic subunit of MgCh (CHLH1) by binding GUN4, a key regulator of MgCh that interacts with CHLH1 (Zhang et al., 2021). These studies reveal the critical role of bilin signaling in photo-acclimation and maintenance of photosynthesis.

The bilin signal transduction pathway has not been elucidated. Identification of key regulatory components involved in bilin signaling and characterization of their biological functions are essential for understanding the mechanistic details of bilin signaling. We employed a plasmid-based method for random insertional mutagenesis to identify *hmox1* suppressors and aimed to isolate components related to bilin signaling. This study describes one such *hmox1* suppressor named *ho1su1*, which grew similar to wild-type *C. reinhardtii* cells in the light. The loss of CHLH1 and PSI-related proteins LHCA1 and PSAD was fully rescued in *ho1su1*, and bilin was surprisingly found to accumulate in *ho1su1* despite the absence of HMOX1. Our study, therefore, reveals the presence of an HMOX1-independent bilin biosynthetic pathway in Chlamydomonas cells.

## Materials and methods

### Chlamydomonas strains and growth conditions

*C. reinhardtii* strains 4A+ (wild-type, mating type plus, mt+), *hmox1* (mt+), *ho1*C2 (genetically complemented strain of *hmox1*), and *hmox2* were described previously (Duanmu et al., 2013). The 4A− (wild-type, mating type minus, mt-) was ordered from the Chlamydomonas Resource Center. The *hmox1* suppressor *ho1su1* (mt+) was generated in this study. All Chlamydomonas strains were maintained on Tris-acetate-phosphate (TAP) plates at 22–25°C in a 20-h dark/4-h light cycle (∼10 μmol photons m^–2^ s^–1^). For growth comparisons, similar numbers of cells resuspended in Tris-phosphate (TP) medium were spotted in TAP medium (mixotrophic growth) or TP medium (phototrophic growth) containing agar and were grown in different light intensities. For total protein extraction and protein accumulation (HMOX1, PSI-related proteins, and CHLH1) analysis, cells were grown in a TAP medium under white light (∼30 μmol photons m^–2^ s^–1^) until they reached a logarithmic phase and were then harvested for protein extraction.

### Generation of *hmox1* suppressors

The *hmox1* cells were resistant to paromomycin due to the insertion of a pBC1 plasmid into the genome (Duanmu et al., 2013) and were cultured in a TAP medium in white light (∼30 μmol photons m^–2^ s^–1^) until they reached a cell density of ∼3 × 10^6^ cells/mL and were harvested by centrifugation at 2,000 *g* for 5 min at 4°C. The cells were resuspended in a TAP medium supplemented with 60 mM sucrose to a density of ∼10^8^ cells/mL and maintained at 4°C for 15 min. Aliquots of 250 μL of cells were transformed with 200–500 ng of *Kpn*I-linearized pHyg3 plasmid containing a hygromycin resistance cassette using electroporation with the BTX Gemini X2 System (1,575 Ω resistance, 50 μF capacitance, and 800 V voltage). Cells were transferred to a TAP medium containing 60 mM sucrose and recovered for 24 h in dim light. Chlamydomonas cells were selected on a solid TP medium supplemented with 10 μg/mL hygromycin in high-intensity light (∼400 μmol photons m^–2^ s^–1^). Survival of transformants containing both hygromycin and paromomycin cassettes accumulated LHCA1, PSAD, and CHLH1 and were also insensitive to high-intensity light were regarded as *hmox1* suppressors.

### Genetic analysis of suppressor *ho1su1*

The *ho1su1* (mt+) was crossed to 4A− (mt−) to generate complete tetrads for genetic analysis and for evaluating the cosegregation of the suppression phenotype with the pHyg3 insertion. In brief, gametes from *ho1su1* and 4A− were induced and mated to form zygotes as described previously (Zhang et al., 2021). Zygotes were transferred to a solid TAP medium in white light (∼30 μmol photons m^–2^ s^–1^) to germinate. The four progeny of the germinating zygote (tetrad) were observed with an inverted microscope and separated using a glass needle. Progeny from complete tetrads were grown on a solid TAP medium and used for phenotypic and genetic analyses.

### Thermal asymmetric interlaced polymerase chain reaction

TAIL-PCR was used to identify genomic sequences flanking the linearized-pHyg3 insertion in *ho1su1* (Dent et al., 2005; Wang et al., 2014). Specific primers (DP4/DP3/DP2 and UP4/UP3/UP2) were designed to amplify the sequences that flank the linearized-pHyg3, both downstream and upstream of the hygromycin resistance gene cassette (see Supplementary Figure 3A) and used along with the degenerate primer RMD227 for primary, secondary, and tertiary rounds of TAIL-PCR reactions. Primers used in this study are listed in Supplementary Table 1. In brief, 20–50 ng genomic DNA from *ho1su1* were used as DNA templates for primary reactions with GoTaq green master mix (Promega, Madison, WI, United States), containing 0.2 μM of specific primer (DP4 or UP4), 1 μM of degenerate primer RMD227, and 10% DMSO. For the second round reaction, the 50-fold diluted primary reaction products were used as DNA templates with GoTaq green master mix, containing 0.2 μM DP3 or UP3, 1 μM RMD227, and 10% DMSO. Then, 1 μL of the 50-fold diluted secondary TAIL-PCR product was used for the tertiary round reactions with GoTaq green master mix, containing 0.2 μM DP2 or UP2, 1 μM RMD227, and 10% DMSO. Parameters for three rounds of TAIL-PCR reactions were set up as previously reported (Dent et al., 2005; Wang et al., 2014). Agarose gel electrophoresis was used for separating TAIL-PCR products. DNA fragments that were extracted from agarose gels were sequenced.

### Plasmid rescue

The plasmid rescue method was also used to identify genomic sequences flanking the linearized-pHyg3 insertion in *ho1su1*. In brief, genomic DNA from *ho1su1* was digested with *Pst*I. Then, the digested genomic DNA was self-ligated using T4 DNA ligase (Thermo Fisher Scientific, Waltham, MA, United States). Competent *E. coli* Tans1-T1 (TransGen, Beijing, China) was transformed with the products of the ligation reaction. Transformants were selected on Luria-Bertani (LB) plates supplemented with 100 μg/mL ampicillin. Single bacterial colonies were cultured and plasmids were extracted and compared using agarose gel electrophoresis and DNA sequence analyses.

### Reverse transcription-PCR

Total RNA was extracted from 4A+, *hmox1*, and *ho1su1* using the TransZol plant kit as recommended by the manufacturer (Transgen, Beijing, China). Contaminating genomic DNA was removed using a DNaseI treatment. RNA was reverse transcribed using an oligo dT primer (Zhang et al., 2018) and the M-MLV reverse transcriptase (Takara, Kusatsu, Shiga, Japan). cDNA obtained from the reverse transcription reaction was used as a template for the amplification of the coding sequence of *HMOX1* using the primers detailed in Supplementary Table 1.

### Rapid amplification of cDNA ends

5′RACE assays were performed to amplify the 5′-end of *HMOX1* transcripts from 4A+, *hmox1*, and *ho1su1* as recommended by a previously published method (Scotto-Lavino et al., 2006). In brief, DNaseI-treated total RNA, primer G1 targeting exon2 of *HMOX1*, and M-MLV reverse transcriptase were used for the reverse transcription of the 5′-end of the *HMOX1* mRNA. The product was ligated to poly(G) at the 5′-end and then used as a template for the 1st round of amplification with primers Qo, Qc, and gene-specific primer G2 targeting exon2 of *HMOX1*. The PCR product was diluted 10-fold and used as a template for further PCR amplification (2nd round) with primer Qi and gene-specific primer G3 spanning exon1 and exon2 of *HMOX1*. The final PCR product was analyzed using agarose gel electrophoresis and sequenced. Alternatively, the final PCR product was ligated into the pEASY-T5 Zero cloning vector (Transgen, Beijing, China) and introduced into competent *E. coli* Tans1-T1. Colonies that yielded PCR products larger than 250 bp when they were analyzed with M13F and M13R primers were used for plasmid extraction. Plasmids were sequenced to confirm they contained cDNA sequences derived from various types of *HMOX1* transcripts from 4A+, *hmox1*, and *ho1su1*.

### Chloroplast expression of a bilin-binding reporter

To evaluate the accumulation of PCB in *hmox1* and *ho1su1*, the cyanobacterial bilin reporter NpF2164g5 from *Nostoc punctiforme* was fused with a twin-strep tag (STII) and inserted into the p322-PsbDp-AtpAp.aadA vector (Duanmu et al., 2013). The resulting plasmid was used for chloroplast transformation experiments with *hmox1* and *ho1su1* using particle bombardment with the PDS-1000/He system (BioRad, California, United States). The bombarded cells were transferred to a solid TAP medium containing 400 μg/mL spectinomycin. Positive transformants that expressed NpF2164g5-STII were identified using PCR and immunoblotting analyses and were further selected with at least 4∼5 rounds of growth on solid TAP medium supplemented with 400 μg/mL spectinomycin to achieve chloroplast DNA homoplasmy. Transgenic lines were named *hmox1:NpF2164g5-STII* and *ho1su1:NpF2164g5-STII*, respectively. NpF2164g5-STII purification was performed using Strep-tag II magnetic beads (Beaver, Suzhou, China) following the protocol recommended by the manufacturer. Purified NpF2164g5-STII protein was concentrated and used for zinc-dependent fluorescence assays to evaluate the presence or absence of covalently bound PCB as described previously (Zhang et al., 2018).

### Expression of truncated HMOX1 and HMOX2 in *hmox1*

The coding region of the truncated *HMOX1* (HMOX1ΔTP, amino acids 61–263, without the predicted chloroplast transit peptide) was fused with the STII tag and cloned into the PsaD-Ble vector. The full-length *HMOX2* genomic DNA was inserted into the pHSP70/RbcS2-Ble vector. The resulting plasmids were digested with *Not*I and *Kpn*I and were introduced into *hmox1* cells using electroporation. Transformants were selected on TAP plates supplemented with 10 μg/mL zeocin. Positive transformants accumulating truncated HMOX1 and HMOX2 were named *hmox1:HMOX1*Δ*TP-STII* and *hmox1:HMOX2*, respectively.

### Protein extraction and immunoblotting analyses

Extraction of total protein from Chlamydomonas and immunoblotting analyses were performed as previously described (Duanmu et al., 2013). Anti-LHCA1 (PHY2510S), anti-STII (PHY1926A), and anti-CHLH1 (PHY5508S) antibodies were obtained from PhytoAB (San Jose, CA, United States). Anti-CP29 and anti-D1 antibodies were obtained from Dr. Jean-David Rochaix (University of Geneva). Anti-HMOX1 and anti-HMOX2 were produced previously (Duanmu et al., 2013). Anti-PSAD (AS09 461) antibodies was purchased from Agrisera (Vännäs, Sweden). All antibodies were used at 1,000-fold dilutions.

### Determination of intracellular reactive oxygen species content in chlamydomonas cells

Chlamydomonas cells were grown under continuous low light (∼30 μmol photons m^–2^ s^–1^) until reaching a logarithmic phase. Then, cells were diluted in TAP medium to a density around 1∼2 × 10^6^ cells/mL and incubated in dark for 24 h. About 2 mL of cell culture were collected by centrifugation and resuspended in an equal volume of TAP medium supplemented with 10 μM 2′,7′-dichlorofluorescein diacetate (DCFH-DA, Sigma Aldrich). Half of the cell cultures were grown in the dark for 30 min, while the other half of the cell cultures were exposed to light (∼200 μmol photons m^–2^ s^–1^) for 30 min. Cells were then washed three times with TAP medium and resuspended in 500 μL of TAP medium. The fluorescence of dichlorofluorescein (DCF) was detected by a microplate reader (Tecan, Männedorf, Switzerland; excitation at 485 nm and emission at 530 nm). Two biological replicates were performed.

## Results

### Isolation of *hmox1* suppressors

**FIGURE 1:**
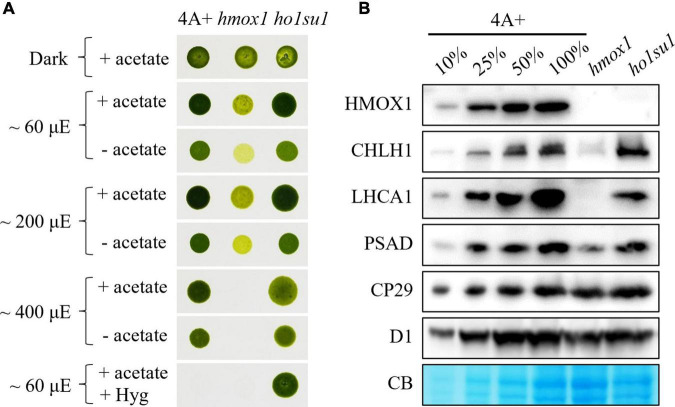
Phenotypic analysis of the *hmox1* suppressor *ho1su1*. **(A)** Mixotrophic (+ acetate) and phototrophic (– acetate) growth of 4A+, *hmox1*, and *ho1su1* in different light intensities. Hyg, hygromycin. **(B)** Immunoblotting analysis of HMOX1, the catalytic subunit of MgCh (CHLH1), representative proteins from PSI (LHCA1 and PSAD) and PSII (CP29 and D1) in 4A+, *hmox1*, and *ho1su1* using specific antibodies. Coomassie blue staining (CB) was used to test for equal loading. Similar amounts of protein were loaded for each genotype. A dilution series (10, 25, and 50%) of total protein from wild-type 4A+ (100%) is provided to help evaluate protein abundance.

To identify key components of a bilin-activated retrograde signaling mechanism, collections of plasmid-based random insertion mutants were screened for mutants with partially or completely recovered phototrophic growth in the photosynthesis-deficient *hmox1* mutant background. As reported previously, phototrophic growth and the accumulation of the catalytic subunit of MgCh and the PSI reaction center and antenna proteins were significantly reduced in *hmox1* relative to wild-type (Duanmu et al., 2013; Wittkopp et al., 2017; Zhang et al., 2021). Indeed, *hmox1* was unable to survive in light intensity of approximately 400 μmol photons m^–2^ s^–1^ (Figure 1A). To screen *hmox1* suppressors, the *Kpn*I-linearized pHyg3 plasmid containing a hygromycin resistance gene cassette was introduced into *hmox1* cells using electroporation, and transformants were selected using phototrophic growth conditions (i.e., TP medium without acetate) at a lethal light intensity for *hmox1* (i.e., approximately 400 μmol photons m^–2^ s^–1^) (Supplementary Figure 1). Transformants that grew in these selective conditions were regarded as candidate suppressors.

Eight candidate *hmox1* suppressors were obtained and characterized. We found that the mixotrophic (with acetate) and phototrophic (without acetate) growth rates of these suppressors in different light intensities were similar to wild-type 4A+. In contrast, increasing light intensities inhibited the growth rate of *hmox1* (Supplementary Figure 2). However, most of these suppressors (i.e., *ho1su2* to *ho1su8*) were not resistant to hygromycin, which indicated that these candidate suppressors lost the hygromycin cassette. The loss of hygromycin resistance was probably due to the low selective concentration of hygromycin, or the instability of hygromycin under this specific harsh condition used for suppressor screening. Only *ho1su1* was resistant to hygromycin and, therefore, presumably contains an intact hygromycin resistance gene in its genome (Supplementary Figure 2 and Figure 1A).

### Rescue of phototrophic growth in *ho1su1*

We next assessed the effect of the suppressor mutation in *ho1su1* on the stability of PSI, photosystem II (PSII), and the catalytic subunit of MgCh (CHLH1). Consistent with previous observations (Wittkopp et al., 2017; Zhang et al., 2021), the levels of CHLH1, the antenna protein (LHCA1), and the reaction center protein (PSAD) of PSI were dramatically reduced in *hmox1* (Figure 1B). In contrast, in *ho1su1*, these proteins accumulated to levels that were similar to wild-type 4A+ (Figure 1B). Furthermore, there were no obvious differences in the levels of the PSII antenna protein CP29 and the core subunit D1 in 4A+, *hmox1*, and *ho1su1* (Figure 1B). These data indicated that the disruption of *HMOX1* and the second site mutations in *ho1su1* had no impact on the accumulation of PSII proteins. These results demonstrate that the phototrophic growth phenotypes of *ho1su1* and 4A+ are indistinguishable and indicate that the suppressor mutation fully restored the photosynthetic deficiency of *hmox1*.

### Co-segregation of the plasmid insert with the suppressor phenotype

**FIGURE 2:**
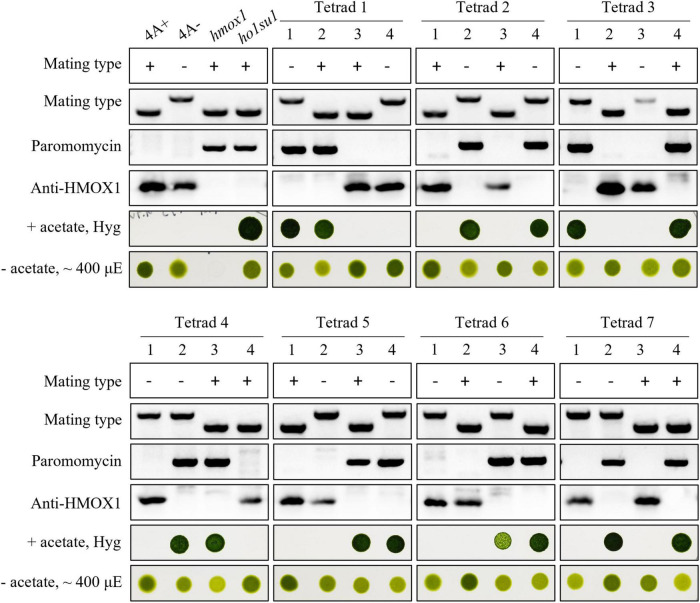
Genetic analyses of *ho1su1*. Progeny from seven complete tetrads derived from crosses between 4A– (mt–) and *ho1su1* (mt+) were used for genetic linkage analysis. Mating type, antibiotic resistance against paromomycin and hygromycin (Hyg), accumulation of HMOX1, and photoautotrophic growth (– acetate) in light intensity of approximately 400 μE were analyzed. PCR amplification of specific DNA fragments was used to test mating type and to test for the presence of the paromomycin resistance gene cassette.

Tetrad analysis was performed to evaluate the genetic linkage between the pHyg3 insertion and the suppressor phenotype in *ho1su1*. Seven complete tetrads from the *ho1su1* (mt+) × 4A− (mt−) cross were isolated and analyzed (Figure 2). Surprisingly, each tetrad contained two progeny that were antibiotic sensitive and two progeny that were resistant to both hygromycin and paromomycin. These data indicate that the pHyg3 insertion and the *HMOX1* gene were located on the same chromosome (Figure 2). Notably, the phototropic growth phenotype of the progeny from these tetrads was similar to wild-type (4A+, 4A−) and *ho1su1*, regardless of the presence or absence of the HMOX1 protein (Figure 2). These results indicate that the pHyg3 insertion is genetically linked to the suppressor phenotype of *ho1su1*.

### Mapping of plasmid insertion sites in *ho1su1*

**FIGURE 3:**
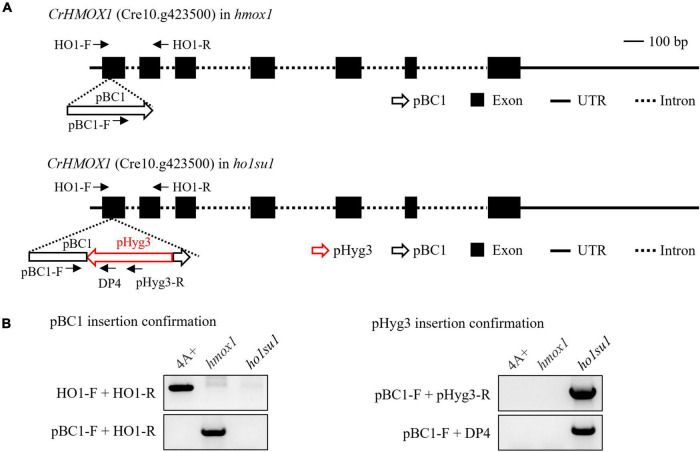
Identification of the pHyg3 insertion site in *ho1su1*. **(A)** Schematic diagram of the paromomycin resistance gene cassette (pBC1) and the hygromycin resistance gene cassette (pHyg3) insertion sites in *hmox1* (upper) and *ho1su1* (lower). The arrows represent primers used for plasmid insert analysis. **(B)** Analysis of pBC1 and pHyg3 insertion sites in *hmox1* and *ho1su1* using PCR and primers depicted in **(A)**.

To identify genomic sequences flanking the pHyg3 insert in *ho1su1*, TAIL-PCR was performed with primers targeting downstream (DP4, DP3, and DP2) and upstream (UP4, UP3, and UP2) regions of the hygromycin resistance gene cassette (Supplementary Figure 3A). We obtained a PCR product downstream of pHyg3 but could not amplify the upstream flanking sequences (Supplementary Figure 3B). The TAIL-PCR product was sequenced and found to match the sequence of the pBC1 insert (i.e., the paromomycin resistance gene cassette) that disrupts *HMOX1* in the *hmox1* mutant (Figure 3A). Indeed, we found that in *ho1su1*, the linearized pHyg3 is inserted exactly in the pBC1 cassette.

Plasmid rescue was also used to determine the genomic sequences flanking the pHyg3 insert in *ho1su1*. The *Pst*I-digested genomic DNA of *ho1su1* was self-ligated and used to transform competent *E. coli* cells (Supplementary Figure 3C). Plasmids were extracted from the five colonies. These plasmids were digested using *Pst*I and plasmid backbones were sequenced (Supplementary Figure 3D). We found that the flanking sequences from these five backbones all matched the sequences from pHyg3 and pBC1, which indicates the presence of both hygromycin and paromomycin cassettes in these plasmids. These data were also confirmed using PCR analyses (Supplementary Figure 3E). These results revealed that the pHyg3 cassette in *ho1su1* was inserted into the pBC1 cassette located in exon 1 of *HMOX1* (Figure 3A). These data were also confirmed using PCR analyses with pBC1- and pHyg3-specific primer pairs (Figure 3B).

### *HMOX1* transcripts in *hmox1* and *ho1su1*

Due to the genetic linkage between the pHyg3 insert and the suppression phenotype of *ho1su1*, we hypothesized that the pHyg3 insertion might create a specific form of the HMOX1 protein and this specific HMOX1 protein could rescue the phototrophic growth of *hmox1*. To this end, we used RT-PCR to test whether *HMOX1* was transcribed in *hmox1* and *ho1su1*. In contrast to 4A+, the full-length *HMOX1* transcript was absent in *hmox1* and *ho1su1* as indicated with PCR assays that utilized the primer pairs F1 + R1. However, truncated *HMOX1* transcripts were found to be present in *hmox1* and *ho1su1* as indicated with PCR assays that utilized the primer pairs F2 + R1 and F3 + R1 (Supplementary Figures 4A,B). The truncated form of the *HMOX1* transcript in *ho1su1* might be translated *in vivo* and accumulated as an enzymatically active protein that rescues the phototrophic-deficient phenotype of *hmox1*.

### Reactivated bilin synthesis in *ho1su1*

**FIGURE 4:**
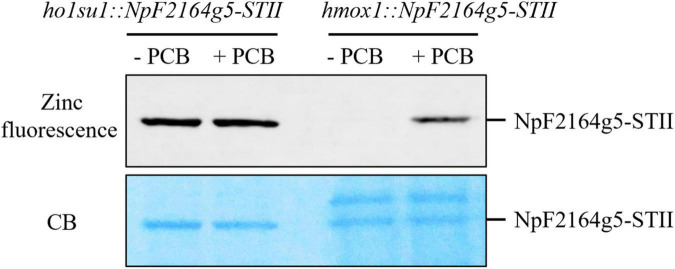
Bilin biosynthesis in *ho1su1*. Cyanobacterial NpF2164g5, a PCB binding reporter, was expressed in the chloroplasts of *hmox1* and *ho1su1*. Affinity purified NpF2164g5-STII protein was assembled with (+ PCB) or without PCB (– PCB) *in vitro* and detected using a zinc-dependent fluorescence assay to test whether a covalently bound PCB was present. Upper panel, zinc fluorescence; lower panel, Coomassie blue staining (CB). The Zinc-dependent fluorescent signal indicates that PCB is covalently bound to the NpF2164g5-STII protein.

It was previously demonstrated that the *hmox1* mutant could not synthesize bilins in the chloroplast and, therefore, was deficient in phototrophic growth (Duanmu et al., 2013; Wittkopp et al., 2017). To determine whether the suppressor phenotype of *ho1su1* was caused by reactivated bilin biosynthesis in the chloroplast, the bilin-binding reporter NpF2164g5 from *Nostoc punctiforme* was expressed in the chloroplast of *hmox1* and *ho1su1*. Affinity-purified NpF2164g5 protein from *hmox1* and *ho1su1* were assembled with or without PCB *in vitro* and tested for the presence or absence of covalently bound PCB using a zinc-dependent fluorescence assay. Consistent with a previous report on the deficiency of PCB biosynthesis in *hmox1* (Duanmu et al., 2013), a fluorescence signal was not detected from the NpF2164g5 protein that was purified from *hmox1* unless exogenous PCB was added to the assay. These data indicate that PCB was absent in the NpF2164g5 protein from *hmox1* (Figure 4). In contrast, we detected fluorescence from the NpF2164g5 protein that was purified from *ho1su1* regardless of whether exogenous PCB was added to the assay (Figure 4). These data indicate that NpF2164g5 binds PCB in *ho1su1* and, therefore, that PCB is synthesized in *ho1su1*.

### Overexpression of HMOX2 and truncated HMOX1 could not rescue the phototrophic growth phenotype of *hmox1*

The accumulation of PCB in *ho1su1* indicates that its precursor BV IXα is synthesized in *ho1su1*. *C. reinhardtii* contains two enzymes that catalyze the conversion of heme to BV IXα, including chloroplastic HMOX1 and cytosolic HMOX2 (Duanmu et al., 2013). Because *HMOX1* was still transcribed in *hmox1* and *ho1su1*, we performed 5′RACE to determine the 5′-end sequences of *HMOX1* transcripts in these two strains (Supplementary Figure 4C). Most of the *HMOX1* cDNA sequences prepared from *hmox1* and *ho1su1* were the same as the *HMOX1* cDNA prepared from 4A+ (shown as black letters in Supplementary Figure 4D), with the exception of approximately 140 bp from the 5′ end (shown as red letters in Supplementary Figure 4D). There was approximately 30 bp that was different at the 5′ end of *HMOX1* in *ho1su1* relative to *hmox1*. Three types of *HMOX1* transcript sequences were confirmed in 4A+, *hmox1*, and *ho1su1* using PCRs with the F0 and R1 primers (Supplementary Figures 4E, 5). Based on the sequences of these transcripts, the only possible open reading frame (ORF) encoding an active heme oxygenase in *ho1su1* might be translated into a truncated HMOX1 protein that contains amino acids 61–263 relative to the HMOX1 protein from 4A+ (shown as black letters on a yellow background in Supplementary Figure 4D) and, therefore, lacks the putative chloroplast transit peptide (i.e., amino acids 1–30).

**FIGURE 5:**
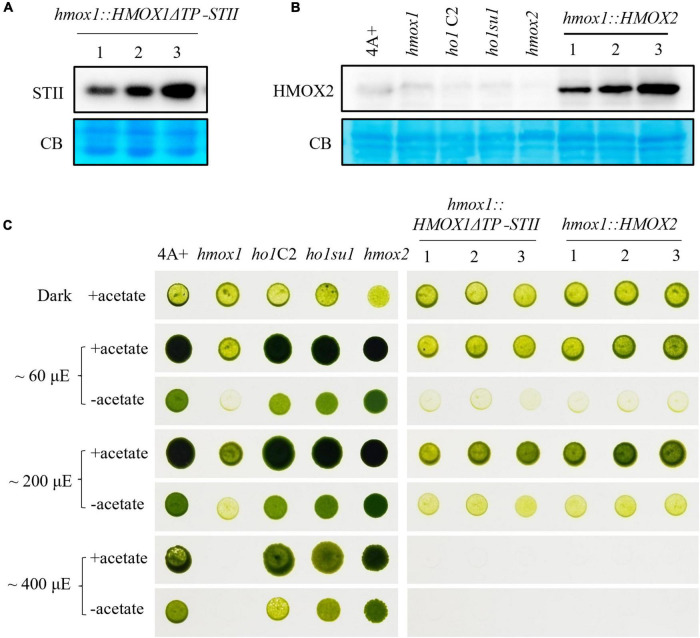
Characterization of *hmox1* expressing either HMOX2 or truncated HMOX1. **(A)** Immunoblotting analysis of *hmox1* expressing HMOX1ΔTP-STII. An anti-STII tag antibody was used. **(B)** Immunoblotting analysis of different strains expressing the HMOX2 protein. Equal amounts of protein extracted from 4A+, *hmox1*, *ho1C2* (*hmox1* cDNA complemented line), *ho1su1*, *hmox2*, and *hmox1::HMOX2* (HMOX2 overexpression lines in *hmox1*) were analyzed using immunoblotting. Anti-HMOX2 antibodies were used. **(C)** Growth of 4A+, *hmox1*, *ho1C2*, *ho1su1*, *hmox2*, *hmox1::HMOX1*Δ*TP-STII*, and *hmox1::HMOX2*. The strains were grown on TAP (with acetate) or TP (without acetate) media at different fluence rates of light.

To test whether the putative-truncated HMOX1 protein could rescue the phototrophic-deficient phenotype of *hmox1*, we expressed the truncated HMOX1 protein containing amino acids 61–263 (hereafter referred to as HMOX1ΔTP) in *hmox1* and selected three independent lines that expressed HMOX1ΔTP (Figure 5A). Growth comparisons showed that expression of truncated HMOX1 could not complement the light-sensitive phototrophic growth deficiency of *hmox1* (Figure 5C).

HMOX2 protein abundance is very low in 4A+ and *hmox1* and is undetectable in immunoblotting assays as previously reported (Duanmu et al., 2013). We used the HSP70/RbcS2 chimeric promoter to constitutively overexpress HMOX2 in *hmox1*. An immunoblotting analysis using HMOX2 antibodies indicated that all three independent *hmox1*::HMOX2 lines accumulated significantly elevated levels of HMOX2 protein (Figure 5B). However, all three lines demonstrated similar degrees of phototrophic growth deficiencies as *hmox1*. These data indicate that the overexpression of HMOX2 could not rescue the *hmox1* phenotype (Figure 5C).

These results ruled out the possibility that the restored bilin biosynthesis and rescued phototrophic growth of *ho1su1* were associated with the overexpression of HMOX2 or the accumulation of a truncated HMOX1. Since HMOX1 and HMOX2 are the only two heme oxygenases that are known to convert heme to BV in *C. reinhardtii*, we conclude that our data have uncovered the existence of an alternative bilin biosynthesis pathway that does not require classical heme oxygenases.

## Discussion

The cyclic tetrapyrrole molecule heme is present in almost all organisms (Larkin, 2016; Sugishima et al., 2019). Heme serves as a cofactor for hemoproteins (cytochromes, hemoglobin, myoglobin, etc.) and performs essential functions in various biological processes (Shimizu et al., 2019; Ryter, 2021). Many organisms utilize heme as an iron source and use heme degrading enzymes to convert heme into pyrrole products, with the concomitant release of iron and carbon monoxide (Kikuchi et al., 2005; Mahawar and Shekhawat, 2018). Several groups of heme oxygenases (HMOXs/HOs) with distinct structures and regiospecificities for heme cleavage have been well-documented (Lyles and Eichenbaum, 2018). When the HO-1 family members (e.g., HMOX1, HemO, and HmuO) degrade heme, they generally yield BV IXα (Wilks and Ikeda-Saito, 2014). In contrast to HO-1, the IsdG family of HOs converts heme to a mixture of formaldehyde and staphylobilin rather than biliverdins (Matsui et al., 2013). PigA and the HO groups containing the HemS motif convert heme into β and δ isoforms of BV (Friedman et al., 2004; Onzuka et al., 2017; Li et al., 2021). Additionally, the HO group belonging to the FMN binding-like superfamily, such as HugZ, HutZ, and HupZ, also converts heme to either β or δ isoforms of BV (Lyles and Eichenbaum, 2018). Among the products formed by the oxidative cleavage of heme, BV IXα is further converted to bilirubin by biliverdin reductase (BVR) in mammals and other types of bilins by FDBRs in aerobic photosynthetic organisms (Tenhunen et al., 1970; Sugishima et al., 2019).

**FIGURE 6:**
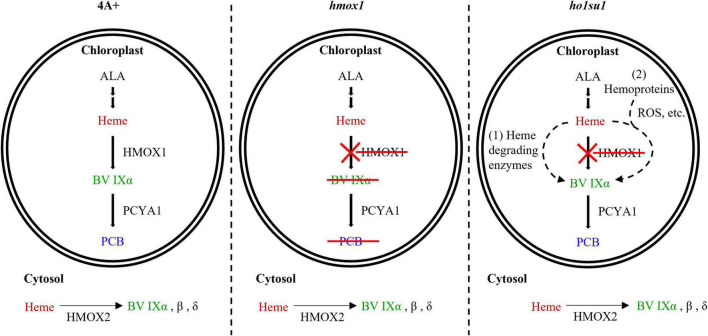
Model for bilin biosynthesis in *ho1su1*. In wild-type 4A+, heme is catabolized by HMOX1 and PCYA1 to yield BV IXα and PCB, respectively (left). In *hmox1*, BV IXα and PCB are not synthesized in the chloroplast due to the disruption of *HMOX1*, although PCYA1 still accumulates (middle). The cytosolic BV IXα produced by overexpressing HMOX2 or truncated HMOX1 is insufficient to support bilin biosynthesis in the chloroplast of *hmox1*. In *ho1su1*, PCB is produced in the absence of HMOX1 (right). Alternative pathways for bilin biosynthesis in *ho1su1* may include (1) the conversion of heme to BV IXα by unknown heme degrading enzymes and (2) reactive oxygen species (ROS) or other metabolites (e.g., ascorbic acid) attacking heme or hemoproteins to produce BV IXα. ALA, 5-aminolevulinic acid.

Bilin biosynthesis is of great importance for photosynthetic organisms because of the essential roles of bilins in light sensing, photon capture, and photoacclimation (Rockwell et al., 2014; Duanmu et al., 2017; Bachy et al., 2022). The Chlamydomonas *hmox1* mutant is deficient in bilin biosynthesis due to the disruption of *HMOX1*, although the PCYA1 protein accumulates at similar levels relative to wild-type 4A+ (Duanmu et al., 2013). The generation of PCB in *ho1su1* indicates that *ho1su1* synthesizes BV IXα from heme. *C. reinhardtii* employs three types of heme oxygenases for heme metabolism, including the chloroplast-localized HMOX1 (Cre10.g423500) that converts heme into BV IXα, the cytosolic HMOX2 (Cre11.g467753) that converts heme into α, β, and δ isoforms of BV, and the IsdG family protein LFO1 (Cre07.g312300) that converts heme into products structurally different from BV (Duanmu et al., 2013; Lojek et al., 2017). BV IXα could be used as a substrate for chloroplast-localized PCYA1 to synthesize PCB. Overexpression of the truncated HMOX1 and HMOX2 could not rescue the phototrophic growth deficiency of *hmox1* (Figure 5), which indicates that the cytosolic BV IXα produced by either the hypothetical truncated HMOX1 or HMOX2 was insufficient to activate the PCB production in the chloroplast. These results provide evidence for an alternative bilin biosynthetic pathway possibly supported by uncharacterized heme degrading enzyme(s) in *C. reinhardtii* (Figure 6).

A previous study demonstrated that Arabidopsis HOZ (At3g03890)—a protein homologous to HugZ—is chloroplast localized and structurally related to non-canonical HMOX and possesses heme degrading activity that yields BV *in vitro* (Wang et al., 2020). The biological function of HOZ in tetrapyrrole metabolism in Arabidopsis requires further characterization. Additionally, there are two proteins that are homologous to HOZ (Cre12.g520200 and Cre02.g098250) in *C. reinhardtii* that may contribute to the degradation of heme and bilin biosynthesis. However, these two genes are not associated with the same chromosome as *HMOX1*. Based on genetic linkage analysis, we excluded the possibility of these two proteins functioning in BV IXα and bilin biosynthesis in *ho1su1*.

In addition to enzyme-catalyzed heme metabolism, heme could also be subjected to non-enzymatic degradation (Nagababu and Rifkind, 2004; Kikuchi et al., 2005). ROS and other metabolites (i.e., ascorbic acid) can catalyze the conversion of heme into various pyrrole products, including BV (Nagababu and Rifkind, 2004). It was reported that hydrogen peroxide (H_2_O_2_) could degrade ferric heme to yield carbon monoxide and BV IXα (Jones et al., 1973). Ascorbic acid randomly attacks the methene bridges of heme and hemoproteins to generate mixtures of four BV isomers (α, β, γ, and δ) (Bonnett and McDonagh, 1973). Our data showed that the *hmox1* mutant contained a much higher level of ROS than 4A+, whereas the ROS content in *ho1su1* is intermediate between *hmox1* and 4A+ (Supplementary Figure 6). Therefore, it is reasonable to hypothesize that heme or hemoproteins in the chloroplast probably undergoes a non-enzymatic degradation process to produce BV IXα, which is further utilized by PCYA1 to generate PCB in *ho1su1* (Figure 6). Further identification of the suppressor gene(s) and investigation of the interactions between its protein product and ROS accumulation and/or heme degradation should be the focus of future work.

Tetrapyrrole biosynthesis and regulation are elegantly controlled to meet the demands of critical cellular processes (Mochizuki et al., 2010). Our study demonstrates that an alternative HMOX1-independent bilin biosynthetic pathway is probably fulfilled by an unknown heme degrading enzyme(s) or non-enzymatic degradation process(es). Identification of genes associated with this alternative pathway should provide deeper insights into elucidating bilin biosynthesis and the regulatory networks of retrograde bilin signaling, which is essential to sustain robust photosynthesis in *C. reinhardtii*.

## Data availability statement

The raw data supporting the conclusions of this article will be made available by the authors, without undue reservation.

## Author contributions

WZ, QF, and DD designed the research and analyzed the results. WZ, RD, WS, and ZL performed the experiments. WZ, RL, and DD wrote and edited the manuscript. All authors read and approved the manuscript.
