# Supplementary material for: Heme oxygenase-independent bilin biosynthesis revealed by a hmox1 suppressor screening in Chlamydomonas reinhardtii
Source: Front Microbiol. 2022 Aug 8;13:956554. doi: 10.3389/fmicb.2022.956554 (PMC9393634; doi:10.3389/fmicb.2022.956554)

Figure S1. Workflow of the *hmox1* suppressor screen.

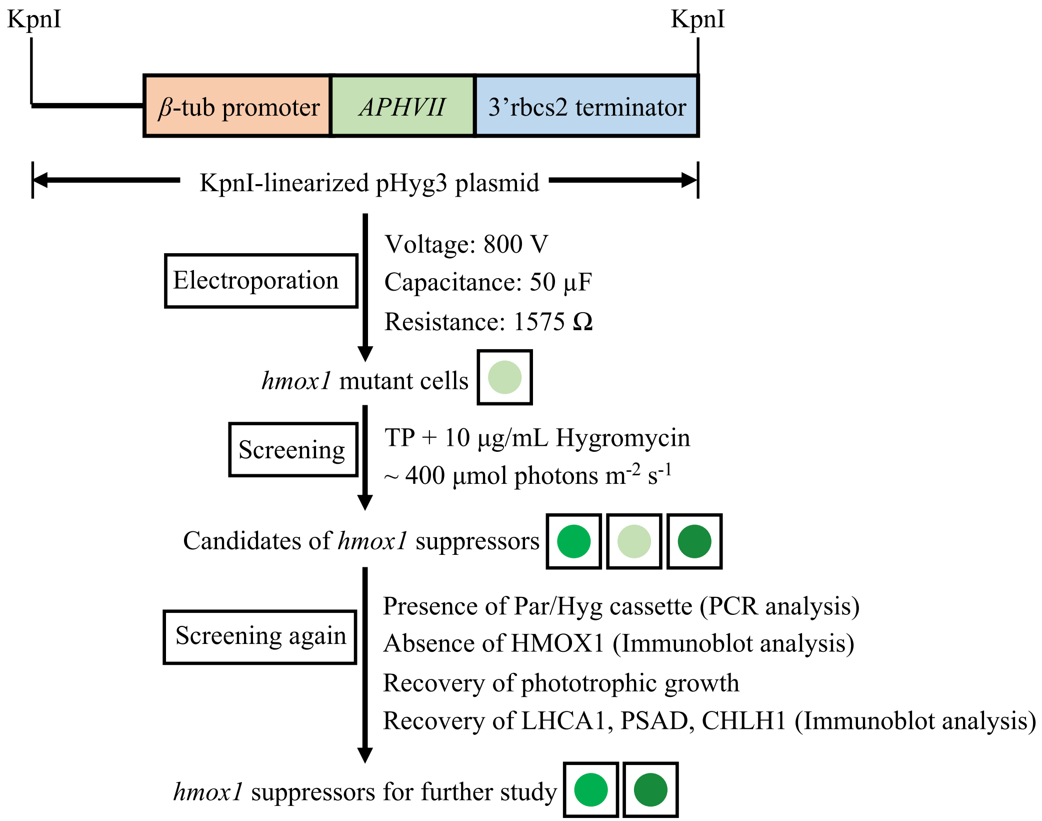

Figure S2. Phototrophic growth comparisons of candidate *hmox1* suppressors.

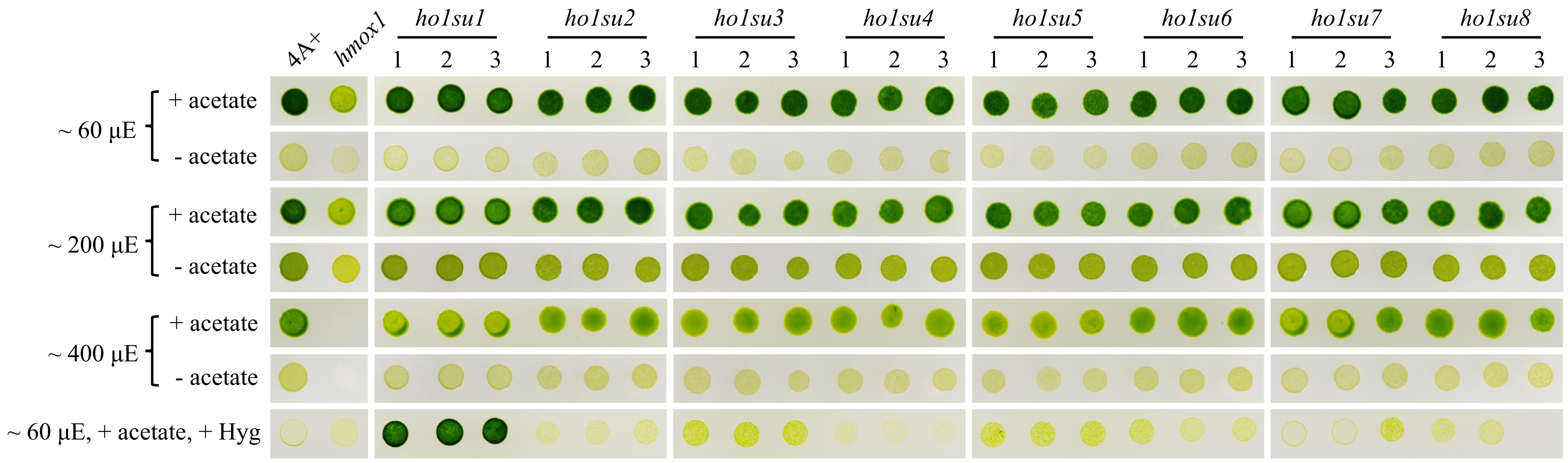

Figure S3. Identification of genomic sequences flanking the pHyg3 insertion in *ho1su1*.

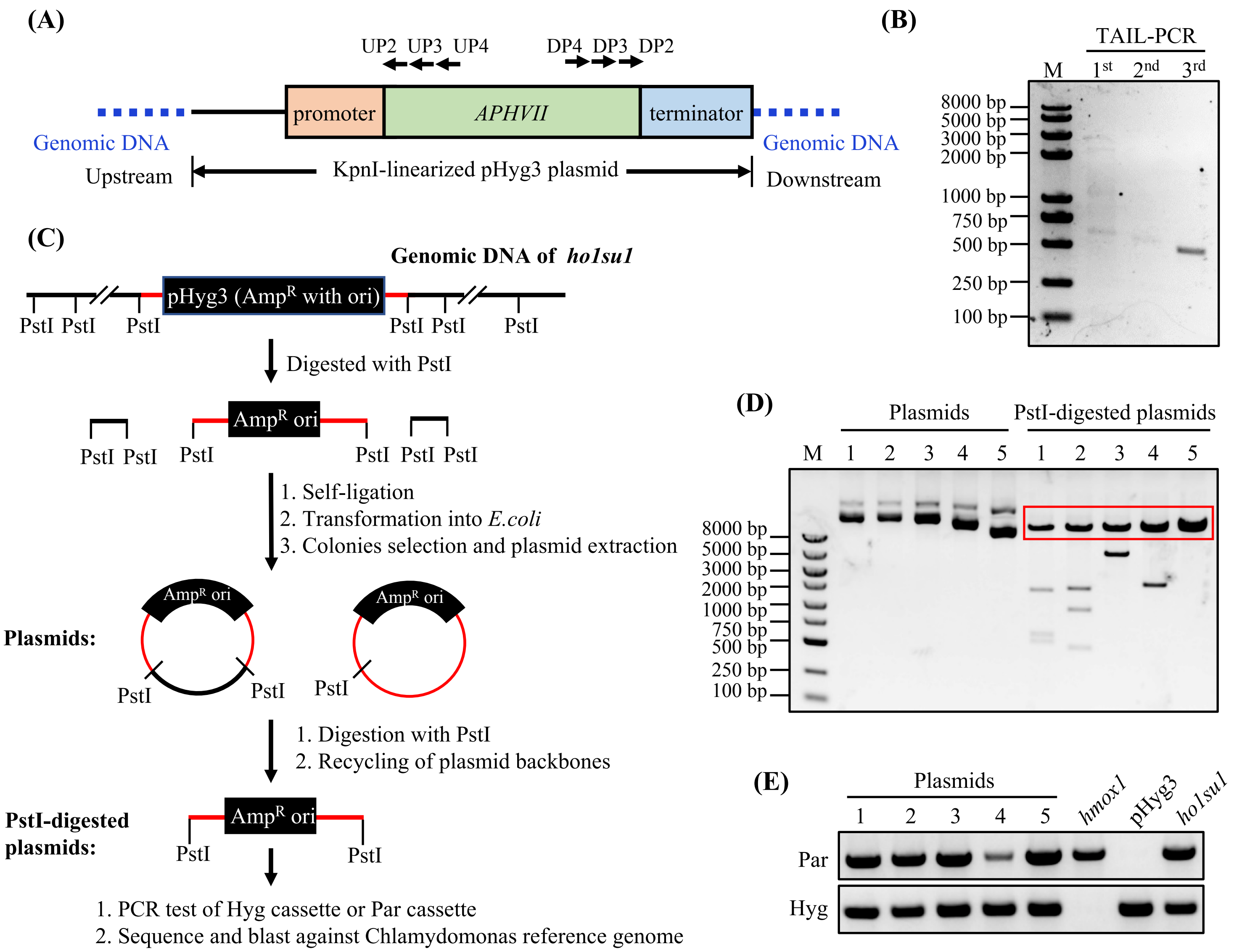

Figure S4. *HMOX1* transcripts in 4A+, *hmox1* and *ho1su1*.

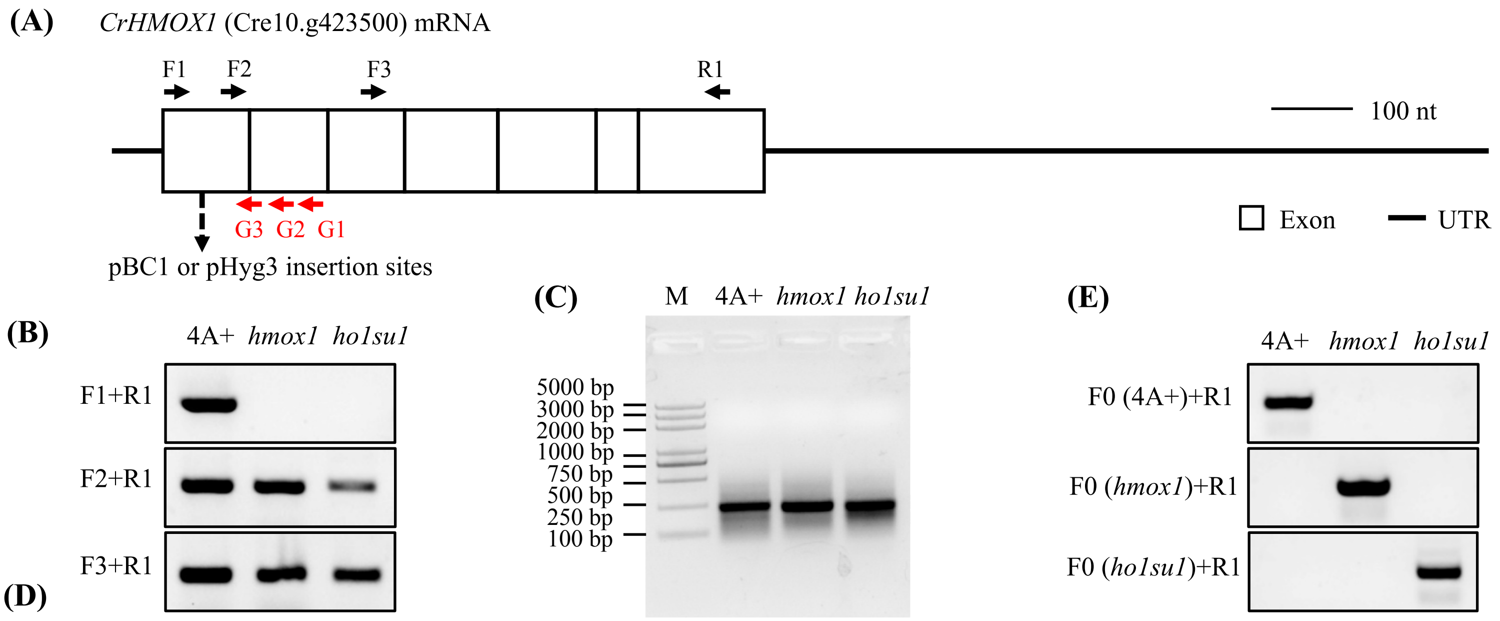

(D)

CrHMOX1 transcript in **4A+**:

ATTTCTCACTCGTTCGATAACAATGCTTCTGTCCTCTCGATCCACCGCCTCGGTCCGTGCCAGCGGTCGCGCTGCACCTGCGCCTCGCCGCGTGGTGCGTGTCCTGGCCCACGGTCATGGT
CACGGAGGTGGCCATGGTCACGGCCATGGCTCGAGTGGCACGGCGGTGCTGACGGAGAAGGACAAGGGCTTTATTGCTGAGATGCGCAAGGTGGCAATGAAGCTGCATACGAAGGACC
AGGCGCCTAAGGAGGGCGAGAAGGAGGCCCCCAAGCAAGGCCCGTGGACGCCCACTCGCCCGGGTTACCTGCGCTTCCTGGTTGAGTCGAAGGAGGTCTTTGATACTTTCGAGCGCCTT
GTCAACTCCAGCGACGCCTACAAGGCGCTGCGCAACACGGGTCTGGAGCGCGGGCCAGGCCTGGCTGCCGACATTGCCTGGATGGAGCAGTCCTTCCAGCTGGCGCCCCCGGACATGA
AGCCGGACGGCGCGGCCGCCACCTACGTGGCCTTTCTGGAGAAGCTCGCCAAGACCGACCCGCCCGCCTTCATCTGCCACTACTACAACTTCTATTTCGCACACACCGCCGGCGGCCGC
ATGATCGGCAACAAGGTGTCCAGCATGCTGCTGGACGGCCAGACCCTGGAGTTCTACAAGTGGCAGGGCGACGTAAACGAGCACCTGGAGGGCGTGCGCAAGTCCATCAACGTGATGG
CCGAGGGCTGGACCCCGGCGGAGAAGGAGCACTGCCTGGCGGAGACCGAGTCCAGCTTCAAGTACAGCGGCCAGATCCTGCGCGCTATCACCGAGGCATAA

CrHMOX1 transcript in ***hmox1***:

AAATCAGTCCTGTAGCTTCATACAAACATACGCACCAATCATGTCAAGCCTCAGCGAGCTCCCCGCTCGAGGTCGACGGTATCGATAAGCTTGATATCGAATTCCTGCAGCCCGGGGGA
TCCACTAGGTCACGGAGGTGGCCATGGTCACGGCCATGGCTCGAGTGGCACGGCGGTGCTGACGGAGAAGGACAAGGGCTTTATTGCTGAGATGCGCAAGGTGGCAATGAAGCTGCAT
ACGAAGGACCAGGCGCCTAAGGAGGGCGAGAAGGAGGCCCCCAAGCAAGGCCCGTGGACGCCCACTCGCCCGGGTTACCTGCGCTTCCTGGTTGAGTCGAAGGAGGTCTTTGATACTT
TCGAGCGCCTTGTCAACTCCAGCGACGCCTACAAGGCGCTGCGCAACACGGGTCTGGAGCGCGGGCCAGGCCTGGCTGCCGACATTGCCTGGATGGAGCAGTCCTTCCAGCTGGCGCCC
CCGGACATGAAGCCGGACGGCGCGGCCGCCACCTACGTGGCCTTTCTGGAGAAGCTCGCCAAGACCGACCCGCCCGCCTTCATCTGCCACTACTACAACTTCTATTTCGCACACACCGCC
GGCGGCCGCATGATCGGCAACAAGGTGTCCAGCATGCTGCTGGACGGCCAGACCCTGGAGTTCTACAAGTGGCAGGGCGACGTAAACGAGCACCTGGAGGGCGTGCGCAAGTCCATCA
ACGTGATGGCCGAGGGCTGGACCCCGGCGGAGAAGGAGCACTGCCTGGCGGAGACCGAGTCCAGCTTCAAGTACAGCGGCCAGATCCTGCGCGCTATCACCGAGGCATAA

CrHMOX1 transcript in ***ho1su1*** :

AAGGGATTTTGGTCATGAGATTATACAAACATACGCACCAATCATGTCAAGCCTCAGCGAGCTCCCCGCTCGAGGTCGACGGTATCGATAAGCTTGATATCGAATTCCTGCAGCCCGGGG
GATCCACTAGGTCACGGAGGTGGCCATGGTCACGGCCATGGCTCGAGTGGCACGGCGGTGCTGACGGAGAAGGACAAGGGCTTTATTGCTGAGATGCGCAAGGTGGCAATGAAGCTGCA
TACGAAGGACCAGGCGCCTAAGGAGGGCGAGAAGGAGGCCCCCAAGCAAGGCCCGTGGACGCCCACTCGCCCGGGTTACCTGCGCTTCCTGGTTGAGTCGAAGGAGGTCTTTGATACTTT
CGAGCGCCTTGTCAACTCCAGCGACGCCTACAAGGCGCTGCGCAACACGGGTCTGGAGCGCGGGCCAGGCCTGGCTGCCGACATTGCCTGGATGGAGCAGTCCTTCCAGCTGGCGCCCCC
GGACATGAAGCCGGACGGCGCGGCCGCCACCTACGTGGCCTTTCTGGAGAAGCTCGCCAAGACCGACCCGCCCGCCTTCATCTGCCACTACTACAACTTCTATTTCGCACACACCGCCGGC
GGCCGCATGATCGGCAACAAGGTGTCCAGCATGCTGCTGGACGGCCAGACCCTGGAGTTCTACAAGTGGCAGGGCGACGTAAACGAGCACCTGGAGGGCGTGCGCAAGTCCATCAACGT
GATGGCCGAGGGCTGGACCCCGGCGGAGAAGGAGCACTGCCTGGCGGAGACCGAGTCCAGCTTCAAGTACAGCGGCCAGATCCTGCGCGCTATCACCGAGGCATAA

Figure S5. Sequence alignment of *HMOX1* transcripts in 4A+, *hmox1* and *ho1su1*.

```
4A+              ATTTCTCACTCGTTCGATAACAATGCTTCTGTCCTCTCGATCCACCGCCTCGGTCCGTGCCAGCGGTCG.CGCTGCACCTGCGCC    84
hmox1         AAATCAGTCCTGTAGCTTCATACAAACATACGCACCAATCATGTCAAGCCTCAGCGA.GCTCCCCGCTCGAGGTCGACGGTATCGATAAGCT    91
ho1su1   AAGGGATTTTGGTCATGAGATTATACAAACATACGCACCAATCATGTCAAGCCTCAGCGA.GCTCCCCGCTCGAGGTCGACGGTATCGATAAGCT    94

4A+      TCGCCGCGTGGTGC.GTGTCCTGGCCCACGGTCATGGTCACGGAGGTGGCCATGGTCACGGCCATGGCTCGAGTGGCACGGCGGTGCTGACGGAG   178
hmox1    TGATATCGAATTCCTGCAGCCCGGGGGATCCACTAGGTCACGGAGGTGGCCATGGTCACGGCCATGGCTCGAGTGGCACGGCGGTGCTGACGGAG   186
ho1su1   TGATATCGAATTCCTGCAGCCCGGGGGATCCACTAGGTCACGGAGGTGGCCATGGTCACGGCCATGGCTCGAGTGGCACGGCGGTGCTGACGGAG   189

4A+      AAGGACAAGGGCTTTATTGCTGAGATGCGCAAGGTGGCAATGAAGCTGCATACGAAGGACCAGGCGCCTAAGGAGGGCGAGAAGGAGGCCCCCAA   273
hmox1    AAGGACAAGGGCTTTATTGCTGAGATGCGCAAGGTGGCAATGAAGCTGCATACGAAGGACCAGGCGCCTAAGGAGGGCGAGAAGGAGGCCCCCAA   281
ho1su1   AAGGACAAGGGCTTTATTGCTGAGATGCGCAAGGTGGCAATGAAGCTGCATACGAAGGACCAGGCGCCTAAGGAGGGCGAGAAGGAGGCCCCCAA   284

4A+      GCAAGGCCCGTGGACGCCCACTCGCCCGGGTTACCTGCGCTTCCTGGTTGAGTCGAAGGAGGTCTTTGATACTTTCGAGCGCCTTGTCAACTCCA   368
hmox1    GCAAGGCCCGTGGACGCCCACTCGCCCGGGTTACCTGCGCTTCCTGGTTGAGTCGAAGGAGGTCTTTGATACTTTCGAGCGCCTTGTCAACTCCA   376
ho1su1   GCAAGGCCCGTGGACGCCCACTCGCCCGGGTTACCTGCGCTTCCTGGTTGAGTCGAAGGAGGTCTTTGATACTTTCGAGCGCCTTGTCAACTCCA   379

4A+      GCGACGCCTACAAGGCGCTGCGCAACACGGGTCTGGAGCGCGGGCCAGGCCTGGCTGCCGACATTGCCTGGATGGAGCAGTCCTTCCAGCTGGCG   463
hmox1    GCGACGCCTACAAGGCGCTGCGCAACACGGGTCTGGAGCGCGGGCCAGGCCTGGCTGCCGACATTGCCTGGATGGAGCAGTCCTTCCAGCTGGCG   471
ho1su1   GCGACGCCTACAAGGCGCTGCGCAACACGGGTCTGGAGCGCGGGCCAGGCCTGGCTGCCGACATTGCCTGGATGGAGCAGTCCTTCCAGCTGGCG   474

4A+      CCCCCGGACATGAAGCCGGACGGCGCGGCCGCCACCTACGTGGCCTTTCTGGAGAAGCTCGCCAAGACCGACCCGCCCGCCTTCATCTGCCACTA   558
hmox1    CCCCCGGACATGAAGCCGGACGGCGCGGCCGCCACCTACGTGGCCTTTCTGGAGAAGCTCGCCAAGACCGACCCGCCCGCCTTCATCTGCCACTA   566
ho1su1   CCCCCGGACATGAAGCCGGACGGCGCGGCCGCCACCTACGTGGCCTTTCTGGAGAAGCTCGCCAAGACCGACCCGCCCGCCTTCATCTGCCACTA   569

4A+      CTACAACTTCTATTTCGCACACACCGCCGGCGGCCGCATGATCGGCAACAAGGTGTCCAGCATGCTGCTGGACGGCCAGACCCTGGAGTTCTACA   653
hmox1    CTACAACTTCTATTTCGCACACACCGCCGGCGGCCGCATGATCGGCAACAAGGTGTCCAGCATGCTGCTGGACGGCCAGACCCTGGAGTTCTACA   661
ho1su1   CTACAACTTCTATTTCGCACACACCGCCGGCGGCCGCATGATCGGCAACAAGGTGTCCAGCATGCTGCTGGACGGCCAGACCCTGGAGTTCTACA   664

4A+      AGTGGCAGGGCGACGTAAACGAGCACCTGGAGGGCGTGCGCAAGTCCATCAACGTGATGGCCGAGGGCTGGACCCCGGCGGAGAAGGAGCACTGC   748
hmox1    AGTGGCAGGGCGACGTAAACGAGCACCTGGAGGGCGTGCGCAAGTCCATCAACGTGATGGCCGAGGGCTGGACCCCGGCGGAGAAGGAGCACTGC   756
ho1su1   AGTGGCAGGGCGACGTAAACGAGCACCTGGAGGGCGTGCGCAAGTCCATCAACGTGATGGCCGAGGGCTGGACCCCGGCGGAGAAGGAGCACTGC   759

4A+      CTGGCGGAGACCGAGTCCAGCTTCAAGTACAGCGGCCAGATCCTGCGCGCTATCACCGAGGCATAA                               814
hmox1    CTGGCGGAGACCGAGTCCAGCTTCAAGTACAGCGGCCAGATCCTGCGCGCTATCACCGAGGCATAA                               822
ho1su1   CTGGCGGAGACCGAGTCCAGCTTCAAGTACAGCGGCCAGATCCTGCGCGCTATCACCGAGGCATAA                               825
```

Figure S6. Comparison of intracellular ROS content during dark-to-light transition.

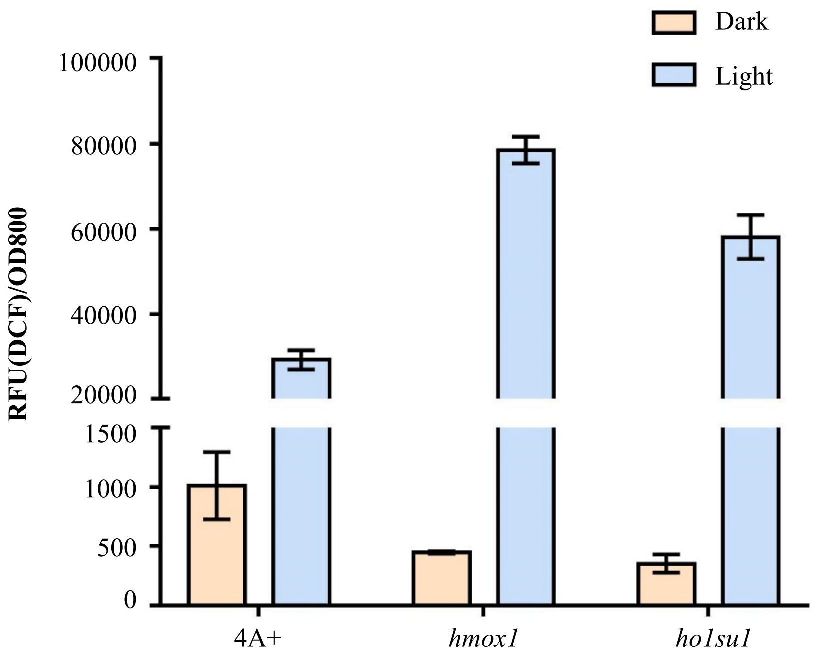

Supplement: Supplementary file 1 [file Data_Sheet_1.PDF]
